# Molecular identification of CTX-M and ^*bla*^OXY/K1 β-lactamase genes in *Enterobacteriaceae *by sequencing of universal M13-sequence tagged PCR-amplicons

**DOI:** 10.1186/1471-2334-9-7

**Published:** 2009-01-22

**Authors:** Hans-Jürg Monstein, Maria Tärnberg, Lennart E Nilsson

**Affiliations:** 1 Clinical Microbiology-LMC, University Hospital S-581 85 Linköping, Sweden; 2 Department of Clinical and Experimental Medicine, Linköping University, S-581 85 Linköping, Sweden; 3 Faculty of Health Sciences, Linköping University, S-581 85 Linköping, Sweden

## Abstract

**Background:**

Plasmid encoded ^*bla*^CTX-M enzymes represent an important sub-group of class A β-lactamases causing the ESBL phenotype which is increasingly found in *Enterobacteriaceae *including *Klebsiella *spp. Molecular typing of clinical ESBL-isolates has become more and more important for prevention of the dissemination of ESBL-producers among nosocomial environment.

**Methods:**

Multiple displacement amplified DNA derived from 20 *K. pneumoniae *and 34 *K. oxytoca *clinical isolates with an ESBL-phenotype was used in a universal CTX-M PCR amplification assay. Identification and differentiation of ^*bla*^CTX-M and ^*bla*^OXY/K1 sequences was obtained by DNA sequencing of M13-sequence-tagged CTX-M PCR-amplicons using a M13-specific sequencing primer.

**Results:**

Nine out of 20 *K. pneumoniae *clinical isolates had a ^*bla*^CTX-M genotype. Interestingly, we found that the universal degenerated primers also amplified the chromosomally located K1-gene in all 34 *K. oxytoca *clinical isolates. Molecular identification and differentiation between ^*bla*^CTX-M and ^*bla*^OXY/K1-genes could only been achieved by sequencing of the PCR-amplicons. *In silico *analysis revealed that the universal degenerated CTX-M primer-pair used here might also amplify the chromosomally located ^*bla*^OXY and K1-genes in *Klebsiella *spp. and K1-like genes in other *Enterobacteriaceae*.

**Conclusion:**

The PCR-based molecular typing method described here enables a rapid and reliable molecular identification of ^*bla*^CTX-M, and ^*bla*^OXY/K1-genes. The principles used in this study could also be applied to any situation in which antimicrobial resistance genes would need to be sequenced.

## Background

Plasmid encoded ^*bla*^CTX-M enzymes represent an important sub-group of class-A β-lactamases which hydrolyse broad-spectrum β-lactam antibiotics causing an extended spectrum β-lactamase (ESBL) phenotype, which is increasingly found in enterobacterial species including *Klebsiella *[1,2]. To date, over 60 different CTX-M-type β-lactamases have been described [3] and divided into five different clusters that reflect similarity at the amino-acid sequence level, namely ^*bla*^CTX-M-1, ^*bla*^CTX-M-2, ^*bla*^CTX-M-8, ^*bla*^CTX-M-9, ^*bla*^CTX-M-25 [2], respectively. More recently, it has been suggested that ^*bla*^CTX-M-45 forms a new, separate cluster [4].

Due to constitutive expression of a chromosomal class A β-lactamases [5], *Klebsiella oxytoca *was shown to have a high level resistance to ceftriaxone and cefotaxime [6]. Originally, this class A β-lactamase was named K1 [7] and later on referred to as KOXY [8] or ^*bla*^OXY [6]. Sequence diversity of the chromosomally located *K. oxytoca *K1-gene and the existence of discrete groups of ^*bla*^OXY-1 and ^*bla*^OXY-2 genes has been described in detail [5,9].

Numerous PCR-based typing assays for the identification of ^*bla*^CTX-M genes have been developed. Initially, detection of all members belonging to specific ^*bla*^CTX-M groups was achieved by combining multiple PCR amplification assays [10,11]. To avoid multiple CTX-M PCR amplification steps, Boyd and co-workers [12] designed a pair of universal, degenerated CTX-M primers, allowing the amplification of hitherto all known ^*bla*^CTX-M genes. However, identification of a ^*bla*^CTX-M genotype at the nucleotide level often required cloning of the PCR-amplicons, followed by DNA sequencing. These methods are labour intensive, time-consuming, expensive and moreover, require a battery of amplicon specific sequencing primers.

In this study, we report on the use of a simple, accurate and universal CTX-M PCR amplification and sequencing assay well suited for high-throughput analysis.

## Methods

### Screening of Klebsiella spp. for cephalosporin resistance during 2001–spring 2007

At the Department of Clinical Microbiology, University Hospital Linköping, susceptibility testing was performed on all *Klebsiella pneumoniae *and *K. oxytoca *clinical isolates. Cefadroxil was used for the screening of cephalosporin resistance which was followed up by testing of resistant isolates with cefotaxime and ceftazidime or direct testing with cefotaxime and ceftazidime [14]. A biochemical panel for identification and differentiation of *Klebsiella *spp. was used. Indole-negative *Klebsiella *spp. clinical isolates were identified as *K. pneumoniae *and indole-positive clinical isolates as *K. oxytoca*.

### Phenotypic ESBL-screening

All cefotaxime and/or ceftazidime resistant clinical isolates were phenotypically screened by Etest using ceftazidime and cefotaxime with and without clavulanic acid (bioMerieux Sverige AB, Askim, Sweden). A reduction of MIC by ≥3 twofold dilutions of the cephalosporin in the presence of clavulanic acid, i. e. a MIC ratio of ≥8 or the presence of phantom- or deformation zones was considered indicative of an ESBL-phenotype. Clinical isolates were stored in glycerol containing Nutrient-broth No 2 (Lab M, Bury, UK) at -70°C until analysis.

### Susceptibility testing of K. oxytoca with K1-genes and K. pneumoniae with ^bla^CTX-M genes

MIC-values for cefotaxime, ceftazidime and piperacillin/tazobactam were determined with Etest (bioMerieux Sverige AB, Askim, Sweden).

### Bacterial type and reference strains

Control strain *K. oxytoca *1980K1 was kindly provided by Dr. D. Livermore, Health Protection Agency, Antibiotic Resistance Monitoring and Reference Laboratory, London, UK. Type strains were purchased from the American Type Culture Collection [15] or the Culture Collection University of Gothenburg [16]; *K. pneumoniae *ATCC 700603, *K. pneumoniae *CCUG 54718, and *K. oxytoca *CCUG 15717^T^.

### Multiple displacement amplification of bacterial DNA

To perform multiple genotyping analysis of our growing collection of CTX-M suspected *K. pneumoniae *and *K. oxytoca *of clinical origin and omitting tedious bacterial culturing, sufficient amounts of bacterial DNA were produced by means of multiple displacement amplification of bacterial DNA [17]. For that purpose, bacteria from frozen cultures (1 μl) and from the reference strains were added to a GenomiPhi-DNA V2 amplification-kit cocktail as recommended by the manufacturer (GE Healthcare Bio-Sciences AB, Uppsala, Sweden).

### Universal ^bla^CTX-M gene PCR amplification

A PCR amplification assay was carried out using 10 pmol of each universal degenerated primer *M13*-CTX-M.U1.SE (*CGTTGTAAAACGACGGCCAGTGA*ATGTGCAGYACCAGTAARGTKATGGC) and CTX-M.U2.AS (TGGGTRAARTARGTSACCAGAAYCAGCGG) targeting the CTX-M and OXY/K1-enzyme genes [modified from 12] and a HotStarTaq-Master mix (Qiagen GmbH, Hilden, Germany) in a final reaction volume of 25 μl using an Applied Biosystems thermo cycler 2720 (Applied Biosystems, Foster City, USA) and 200 μl thin-walled reaction tubes. This yields an approximately 600 bp PCR amplicon corresponding to 68% of the CTX-M and OXY/K1-enzyme encoding nucleotide sequences.

PCR amplification conditions were as follows: initial denaturation step at 95°C for 15 min; 30 cycles of denaturation at 95°C for 30 s; annealing at 55°C for 30 s; extension at 72°C for 2 min, and a final extension step at 72°C for 10 min. Subsequently, PCR-amplicons were separated electrophoretically on a precast 2% agarose E-gel (Invitrogen, Carlsbad, CA, USA).

### DNA sequence analysis

DNA sequence analysis of M13-sequence tagged CTX-M PCR-amplicons was carried out using a M13 uni (-21) primer by a customer DNA sequencing service (Eurofins MWG Operon GmbH, Martinsried, Germany). Prior to DNA sequencing, PCR-amplicons were treated with ExoSAP-IT to inactivate excess of oligonucleotide primers, following the supplier's protocol (USB Europe GmbH, Staufen, Germany). The PCR products were then lyophilised and sequenced. Generated DNA sequences were aligned, edited and compared with ^*bla*^CTX-M DNA and ^*bla*^CTX-M-like DNA sequences using the CLC bioinformatics freeware v.3.2.3 [18]. ^*bla*^CTX-M, ^*bla*^OXY, K1, and K1-like DNA sequences were retrieved from the NCBI Entrez Nucleotide database [19].

### *In silico *DNA sequence analysis

**Figure 1:**
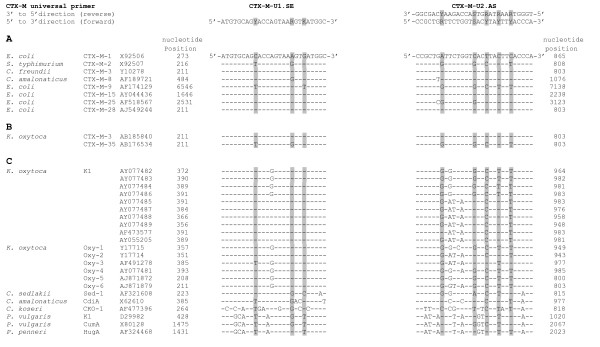
**Partial DNA sequence alignment of (A) CTX-M type sequences *E. coli *CTX-M 1 (Group 1), *E. coli *CTX-M-9 (Group 9), *E. coli *CTX-M-15 (Group 1), *E. coli *CTX-M-25 (Group 25), *E. coli *CTX-M-28 (Group 2), *S. typhimurium *CTX-M-2 (Group 2), *C. freundii *CTX-M-3 (Group 1), *C. amalonaticus *CTX-M-8 (Group 8); **(B)**, *K. oxytoc*a CTX-M-3 (Group 1) and *K. oxytoc*a CTX-M-35 (Group 2), **(C) **chromosomally CTX-M-like sequences *K. oxytoca *K1, *K. oxytoca *OXY-1 to OXY-6, *C. sedlakii *Sed-1, *C. amalonaticus *CdiA, *C. koseri *CKO, *P. vulgaris *K1, *K. vulgaris *CumA, and *P. penneri *HugA genes and the universal degenerated CTX-M-U1.SE and CTX-M-U2**. AS primers. Dashes indicate sequence homologies. The degenerated nucleotide sequence positions in the primers and its corresponding nucleotides in the aligned genes are indicated in grey. For clarity, sense and antisense DNA sequences of the universal degenerated CTX-M-U2.AS (reverse) primer are given.

**Figure 2:**
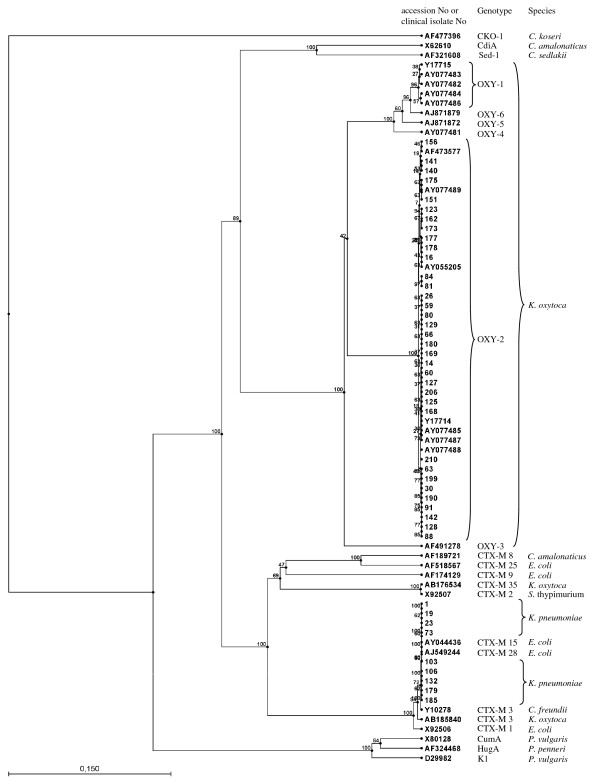
**Phylogenetic tree from partial *Enterobacteriaceae *^*bla*^CTX-M and K1-like DNA sequences**. *K. pneumoniae *^*bla*^CTX-M-15/28 and *K. oxytoca *K1/OXY-2 form distinct cluster groups, distinguishable from the K1/OXY-1 cluster, respectively. *P. penneri *HugA, *P. vulgaris *CumA and K1, *C. sedlakii *and *C. amalonaticus *form separate clades.

^*bla*^CTX-M type strains *E. coli *^*bla*^CTX-M-1 [GenBank:X92506] (^*bla*^CTX-M Group 1), *E. coli *^*bla*^CTX-M-9 [GenBank:AF189721] (^*bla*^CTX-M Group 9), *E. coli *^*bla*^CTX-M-15 [GenBank:AY044436] (^*bla*^CTX-M Group 1), *E. coli *^*bla*^CTX-M-25 [GenBank:AF518567] (^*bla*^CTX-M Group 25), *E. coli *^*bla*^CTX-M-28 [GenBank:AJ549244] (^*bla*^CTX-M Group 25), *Salmonella *Typhimurium ^*bla*^CTX-M-2 [GenBank:X925079] (^*bla*^CTX-M Group 2), *Citrobacter freundii *^*bla*^CTX-M-3 [GenBank:Y10278] (^*bla*^CTX-M-Group 1), *Citrobacter amalonaticus *^*bla*^CTX-M-8 [GenBank:AF189721] (^*bla*^CTX-M Group 8), *K. oxytoca *K1-genes [GenBank:AY077482–AY077489, AF473577, AY055205], *K. oxytoca *^*bla*^OXY-1 [GenBank:Y17715], ^*bla*^OXY-2 [GenBank:Y17714], ^*bla*^OXY-3 [GenBank:AF491278], ^*bla*^OXY-4 [GenBank:AY077481], ^*bla*^OXY-5 [GenBank:AJ871872] and ^*bla*^OXY-6 [GenBank:AJ871879], *K. oxytoca *^*bla*^CTX-M-3 [GenBank:AB185840] (^*bla*^CTX-M Group 1) and ^*bla*^CTX-M-35 [GenBank:AB176534] (^*bla*^CTX-M Group 1), chromosomally encoded β-lactamase genes from *Citrobacter sedlakii *Sed-1 [GenBank:AF321608], *Citrobacter amalonaticus *CdiA [GenBank:X62610], *Citrobacter koseri *CKO [GenBank:AF477396], *Proteus vulgaris *K1 [GenBank:D29982], *Proteus vulgaris *CumA [GenBank:X80128], *Proteus penneri *HugA [GenBank:AF324468] and the universal degenerated primers CTX-M.U1.SE and CTX-M.U2.AS, respectively (Fig. 1), were aligned using ClustalW [20]. A dendrogram consisting of all partial DNA sequences used for primer comparison in figure 1 and DNA sequences derived from the clinical isolates was constructed using the CLC bioinformatics freeware v.3.2.3 [18] and UPGMA clustering (Fig. 2). DNA sequences were edited to comprise the relevant DNA sequences between the two universal degenerated CTX-M primers (Fig. 1).

## Results

### Distribution of *Klebsiella *spp

The number of *K. pneumoniae *and *K. oxytoca *clinical isolates of each year was in the range 457 to 864 (*K. pneumoniae*) and 270 to 455 (*K. oxytoca*), respectively, comprising 99% of the genus *Klebsiella *collected and analysed at the Department of Clinical Microbiology, University Hospital, Linköping, Sweden.

### Screening of cephalosporin resistance and ESBL-phenotype

The cefadroxil resistance rate was in the range of 1.5% to 3.2% for *K. pneumoniae *and 3.7% to 6.4% for *K. oxytoca*. Twenty *K. pneumoniae *and 34 *K. oxytoca *clinical isolates were selected from a positive ESBL-phenotypic screening during 2001 to spring 2007. All *K. oxytoca *isolates (n = 34) revealed an ESBL-phenotype by screening with Etest using cefotaxime with and without clavulanic acid. All isolates were negative in the corresponding test with ceftazidime. Similarly, a majority of *K. pneumoniae *isolates (16 of 20) revealed an ESBL-phenotype in both ESBL Etests. Two of the *K. pneumoniae *isolates revealed an ESBL-phenotype only with cefotaxime and two isolates only with ceftazidime, respectively.

### ^*bla*^CTX-M PCR amplification and partial DNA sequence analysis

DNA sequencing of M13-sequence-tagged universal CTX-M PCR-amplicons of MDA-DNA derived from *K. pneumoniae *and *K. oxytoca *of clinical origin revealed the presence of ^*bla*^CTX-M genes in 9 out of 20 *K. pneumoniae *and the presence of the K1-gene in all 34 *K. oxytoca *clinical isolates. According to the phylogenetic tree constructed from partial ^*bla*^CTX-M, ^*bla*^OXY and K1-DNA sequences located between the two universal degenerated primers (Fig. 1), nine *K. pneumoniae *clinical isolates formed a unique cluster with *E. coli *^*bla*^CTX-M-15, [GenBank:AY044436] and *E. coli *^*bla*^CTX-M-28 [GenBank:AJ549244] which itself is closely related to the *C. freundii *CTX-M-3 [GenBank:Y10278], *K. oxytoca *^*bla*^CTX-M-3 [GenBank:AB185840], and *E. coli *^*bla*^CTX-M-1 [GenBank:X92506] cluster, respectively (Fig. 2). Similarly, the *K. oxytoca *K1 clinical isolates form a unique K1/^*bla*^OXY-2 cluster together with *K. oxytoca *K1 [GenBank:AF473577, AY077489, AY055205, AY077487, AY077485, and AY077488] and *K. oxytoca *^*bla*^OXY-2 [GenBank:Y17714], respectively (Fig 2). None of the *K. oxytoca *K1 clinical isolates clustered within the K1/^*bla*^OXY-1 cluster. Clearly, partial DNA sequence analysis of the CTX-M PCR-amplicons did not allow an unequivocal discrimination of the ^*bla*^CTX-M genes. However, our data indicate the presence of a ^*bla*^CTX-M-15/28 genotype in the *K. pneumoniae *clinical isolates.

### Susceptibility testing of *K. oxytoca *with K1-genes and *K. pneumoniae *with ^bla^CTX-M genes

The MIC-values for cefotaxime and ceftazidime for the *K. oxytoca *isolates were in the range of 0.5 to 8 mg/l and 0.125 to 4 mg/l, respectively. Corresponding MIC-values for the *K. pneumoniae *isolates with CTX-M genotypes were in the range of 64 to 256 mg/l and 16 to 256 mg/l, respectively. The susceptibility for piperacillin/tazobactam was lower in the *K. oxytoca *isolates with MIC-values ≥128 mg/l compared to MIC-values between 4 to 64 mg/l for the *K. pneumoniae *isolates.

### *In silico *DNA sequence comparison

The finding that the universal degenerated CTX-M primer-pair amplified the chromosomally located K1-enzyme in *K. oxytoca *prompted us to perform a DNA sequence alignment of the universal CTX-M primer-pair with *Enterobacteriaceae *^*bla*^CTX-M, ^*bla*^OXY, K1, and K1-like genes retrieved from the Entrez Nucleotide database (Methods). As illustrated in figure 1, the universal degenerated CTX-M primers revealed a high degree of DNA sequence similarity between the target sequences present in the *E. coli, S. *Typhimurium, *C. freundii and C. amalonaticus *^*bla*^CTX-M type-gene; *K. oxytoca *^*bla*^CTX-M-3 and ^*bla*^CTX-M-35 genes, *K. oxytoca *K1 and ^*bla*^OXY-1 to ^*bla*^OXY-6 genes; the chromosomally encoded *C. sedlakii *Sed-1 and *C. amalonaticus *CdiA showed a lower degree of sequence similarity compared to *C. koseri *CKO, *P. vulgaris *K1, *P. vulgaris *CumA, and *P. penneri *HugA genes, respectively. With the exception of *C. koseri CKO, P. vulgaris *K1, *P. vulgaris *CumA and *P. penneri *HugA DNA sequences, most of the nucleotide variations are observed at 5'-Y, R, K-3' positions in primer CTX-M-U1.SE and 5'-R, R, R, S, Y-3' positions (were R stands for purine, Y stands for pyrimidine, S stands for G or C, and K stands for G or T) in primer CTX-M-U2.AS (Fig. 1). The GC-rich 3'-ends of the primers are highly conserved within the corresponding ^*bla*^CTX-M, ^*bla*^OXY, and K1 target DNA sequences. This may explain why the universal degenerated CTX-M primer-pair amplified ^*bla*^CTX-M and K1 sequences.

## Discussion

The increased prevalence of *Enterobacteriaceae *that produce ^*bla*^CTX-M enzymes makes new demands on clinical routine microbiology laboratories to perform ^*bla*^CTX-M typing. Due to the growing number of ^*bla*^CTX-M enzymes, the traditional iso-electrofocusing appears not to be the method of choice for establishing an enterobacterial ^*bla*^CTX-M genotype any longer. Molecular techniques for identification and classification of ^*bla*^CTX-M genes in clinical isolates on a large scale have been described. Recently, a multiplex CTX-M PCR (MP-PCR) amplification assay was described which allows differentiation between different ^*bla*^CTX-M subtype groups [21]. However, using this particular MP-PCR assay, we often observed non-specific PCR amplification in *K. oxytoca *isolates. Subsequent cloning and DNA sequencing analysis revealed that the unspecific PCR-amplicons represented K1-enzyme gene sequences. Thus, misinterpretation of strains as active ^*bla*^CTX-M producers based on false positive PCR amplification might cause false reporting of ^*bla*^CTX-M genes in *K. oxytoca*.

A different approach has been used by Galas and co-workers [22]. These authors described the use of a CTX-M-consensus primer-pair to establish a ^*bla*^CTX-M-genotype in *Enterobacteriaceae*, including *K. oxytoca *[22]. However, *in silico *DNA sequence analysis reveals that this consensus CTX-M primer-pair [23] also targets ^*bla*^OXY/K1-genes such as *K. oxytoca *^*bla*^OXY-2 [Genbank:Y17714] at positions 348–367 (MA1 primer) and 872–391 (MA2 primer), and K1 gene [GenBank:AY077482] at positions 369–378 (MA1 Primer) and 893–911 (MA2 primer), respectively. Thus, based on PCR amplification alone, it seems to be far from clear whether these *K. oxytoca *isolates would have a ^*bla*^CTX-M, or a ^*bla*^OXY/K1-genotype, respectively. This question can only be settled by DNA-sequencing of the PCR amplicons.

The use of M13-sequence tagged PCR-amplicons in combination with M13-specific sequencing primers was originally described for sequencing of *Staphylococcus aureus *protein A (Spa-typing) PCR-amplicons [24]. Our results employing the same technique for sequencing of β-lactamase PCR amplicons convincingly demonstrate that that the use of a M13-sequence tagged CTX-M.U1.SE primer allowed for an unequivocal discrimination of ^*bla*^CTX-M and K1-genes. Moreover, our results support our previous findings indicating that ^*bla*^CTX-M and K1-enzyme genes might have some degree of sequence similarity [13]. Extended *in silico *analysis furthermore revealed a high degree of sequence similarities between *Enterobacteriaceae *^*bla*^CTX-M, ^*bla*^OXY 1–6, and K1 related sequences (Fig. 1), respectively. Thus, based on PCR amplification alone using universal degenerated CTX-M primer-pairs, it would be difficult, not to say impossible to distinguish between an *Enterobacteriaceae *^*bla*^CTX-M or K1/OXY genotype if sequencing had not been performed.

Fournier and co-workers [8] have described the existence of two discrete groups of *K. oxytoca *^*bla*^OXY-1 and ^*bla*^OXY-2 enzymes. Later on, it was shown that ^*bla*^OXY-1 and ^*bla*^OXY-2 genes are expressed in two genetic *K. oxytoca *groups, namely *K. oxytoca *strain SG266 and SG271, respectively [25]. So far, six groups of OXY β-lactamases have been identified and characterised in *K. oxytoca *[26]. In our study we have found that all *K. oxytoca *clinical isolates form a distinct *K. oxytoca *K1/OXY-2 cluster group distinguishable from the K1/OXY-1 and the *K. pneumoniae *CTX-M15/28 cluster group, respectively. Moreover, the phylogenetic tree that was established implies the existence of a chromosomally located β-lactamase super-gene family in *Enterobacteriaceae *(Fig. 2). This is in agreement with previous reports describing that chromosomally encoded class A β-lactamases found in *Klebsiella *species are highly conserved at the amino-acid level compared to class A β-lactamases found in other *Enterobacteriaceae *[27-31]. The *Citrobacter *spp. DNA sequences included in the phylogenetic tree form separate clades. This is in agreement with earlier reports showing that *C. koseri *CKO-1 and *C. amalonaticus *CdiA isolates carry highly divergent β-lactamase genes despite the fact that they show a highly similar biochemical profile and 16S rDNA sequence similarity [32]. Biochemical methods may not always be adequate to identify *Klebsiella *spp. and their phylogenetic groups in clinical microbiology laboratories because several species share similar biochemical profiles [33,34]. Therefore, molecular techniques as applied in the present study may help to accomplish bacterial genotyping at reasonable costs and time.

MIC-value determination of piperacillin/tazobactam in this study also distinguished between *K. oxytoca *with K1 β-lactamase from CTX-M producing *K. pneumoniae*, showing higher MIC-values. Furthermore, the MIC-values for cefotaxime and ceftazidime for *K. oxytoca *with K1-genes was lower than for CTX-M producing *K. pneumoniae*. Similar results have been reported by Potz and co-workers [35].

## Conclusion

The PCR-based molecular typing method described here enables a rapid and reliable identification of CTX-M and OXY/K1-genes. The principles used in the present study can be applied to any situation in where antimicrobial resistance genes are to be sequenced. This is desirable because only sequencing of full-length reading frames will allow for an unequivocal discrimination between various subtypes of antimicrobial resistance genes such as ^*bla*^CTX-M, ^*bla*^SHV and ^*bla*^TEM gene-families, respectively. Moreover, the use of M13-sequence tagged primers in PCR amplification assays facilitates amplicon sequencing since only one single sequencing primer (M13) is required.

## Competing interests

The authors declare that they have no competing interests.

## Authors' contributions

HJM, MT and LEN participated in the conception, design, drafting of the manuscript, and final approval of the version to be published. HJM and MT were responsible for the acquisition, analysis and interpretation of the molecular biology based data. MT and LEN were responsible for the clinical strain collection and phenotypic screening, analysis and interpretation of phenotypic data.

## Pre-publication history

The pre-publication history for this paper can be accessed here:

http://www.biomedcentral.com/1471-2334/9/7/prepub
